# Supplementary figures and images for: Coral histology reveals consistent declines in tissue integrity during a marine heatwave despite differences in bleaching severity
Source: PeerJ. 2025 Jan 3;13:e18654. doi: 10.7717/peerj.18654 (PMC11702357; doi:10.7717/peerj.18654)

*Montipora capitata*

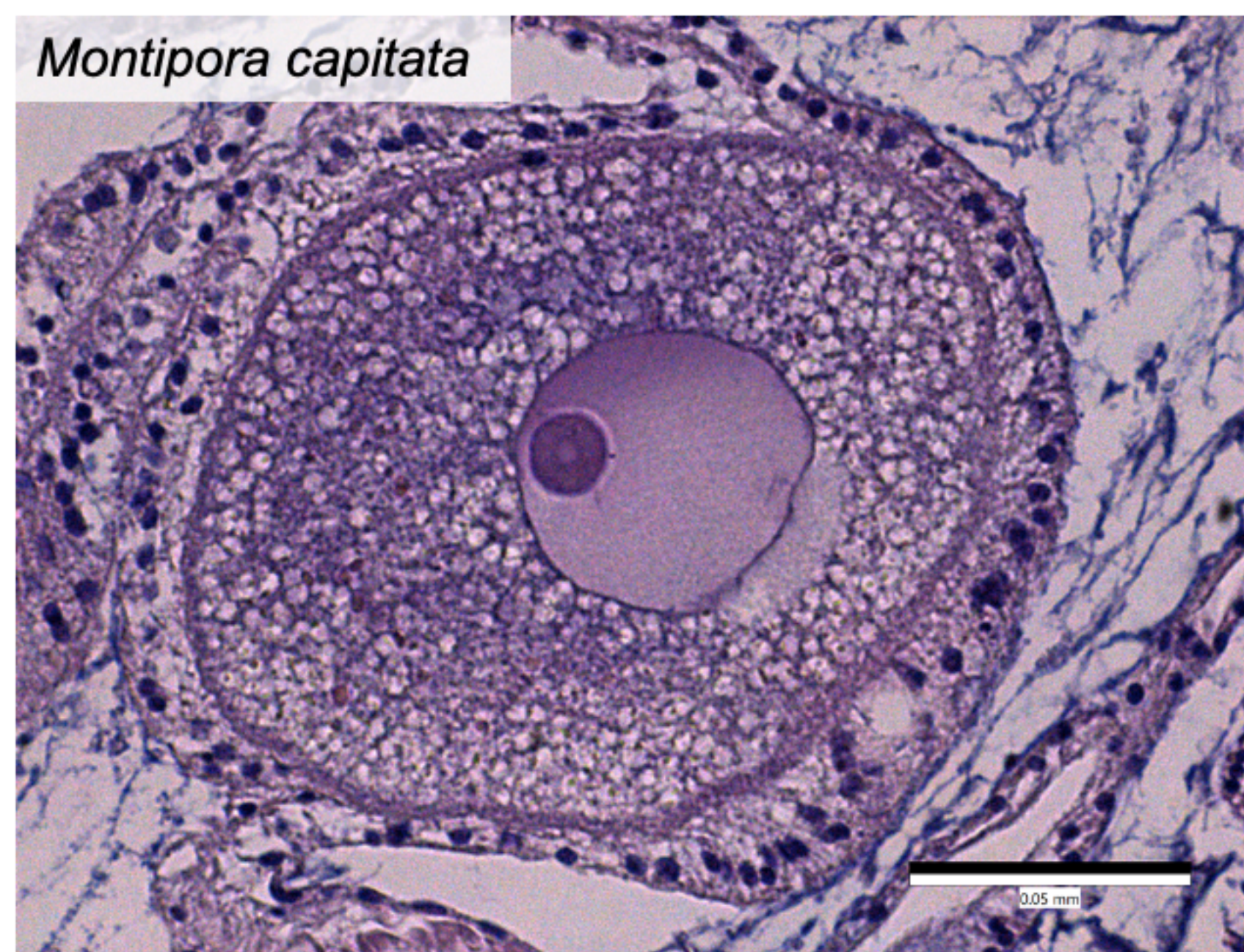

*Porites compressa*

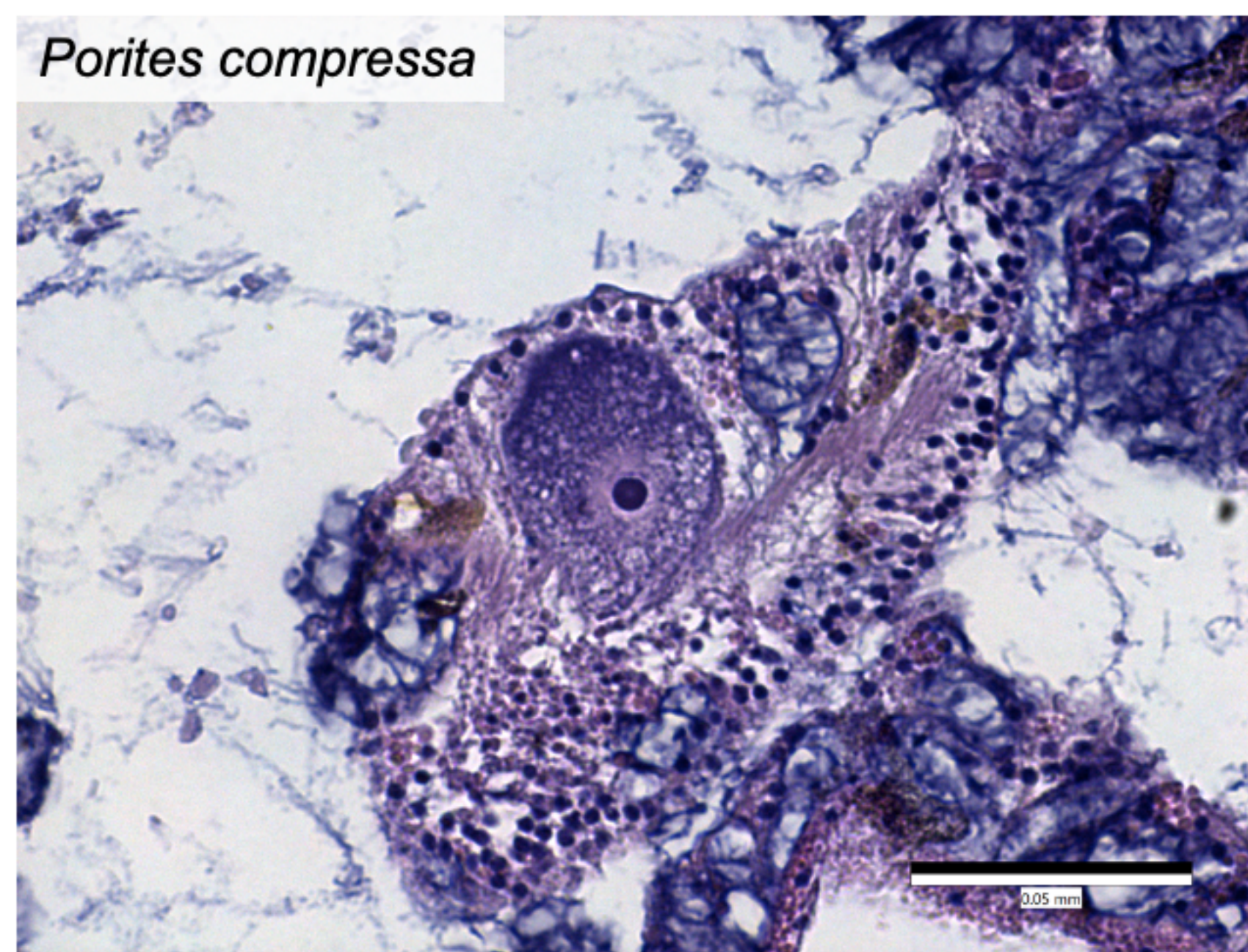

Supplement: Supplemental Information 1 — Left image shows a Stage II oocyte with a clear nucleus and darker nucleolus on the left edge of the nucleus. Right image shows a Stage I oocyte from a Porites compressa colony with a faint nucleus and darker nucleolus in the center. Scale bars represent 50 um. [file peerj-13-18654-s001.pdf]

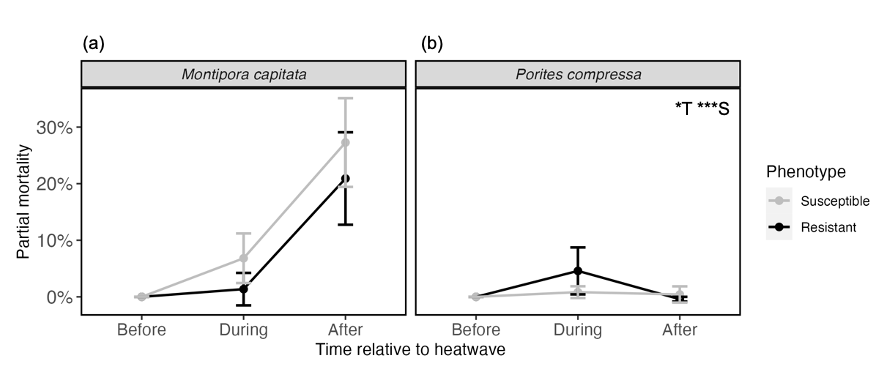

Supplement: Supplemental Information 2 — (A–B) Tissue loss (mean ± SE) as a percentage of the entire colony compared to its original size in July 2019. Percentages are cumulative and decreased partial mortality represents tissue regrowth. Bleaching-susceptible means are in gray and bleaching-resistant means are in black. Inset indicates statistical significance (∗∗∗p < 0.0001) of species (S) and time (T). Underwater images were taken before (July 2019), during (October 2019), and two years after (March 2022) the 2019 marine heatwave. [file peerj-13-18654-s002.png]

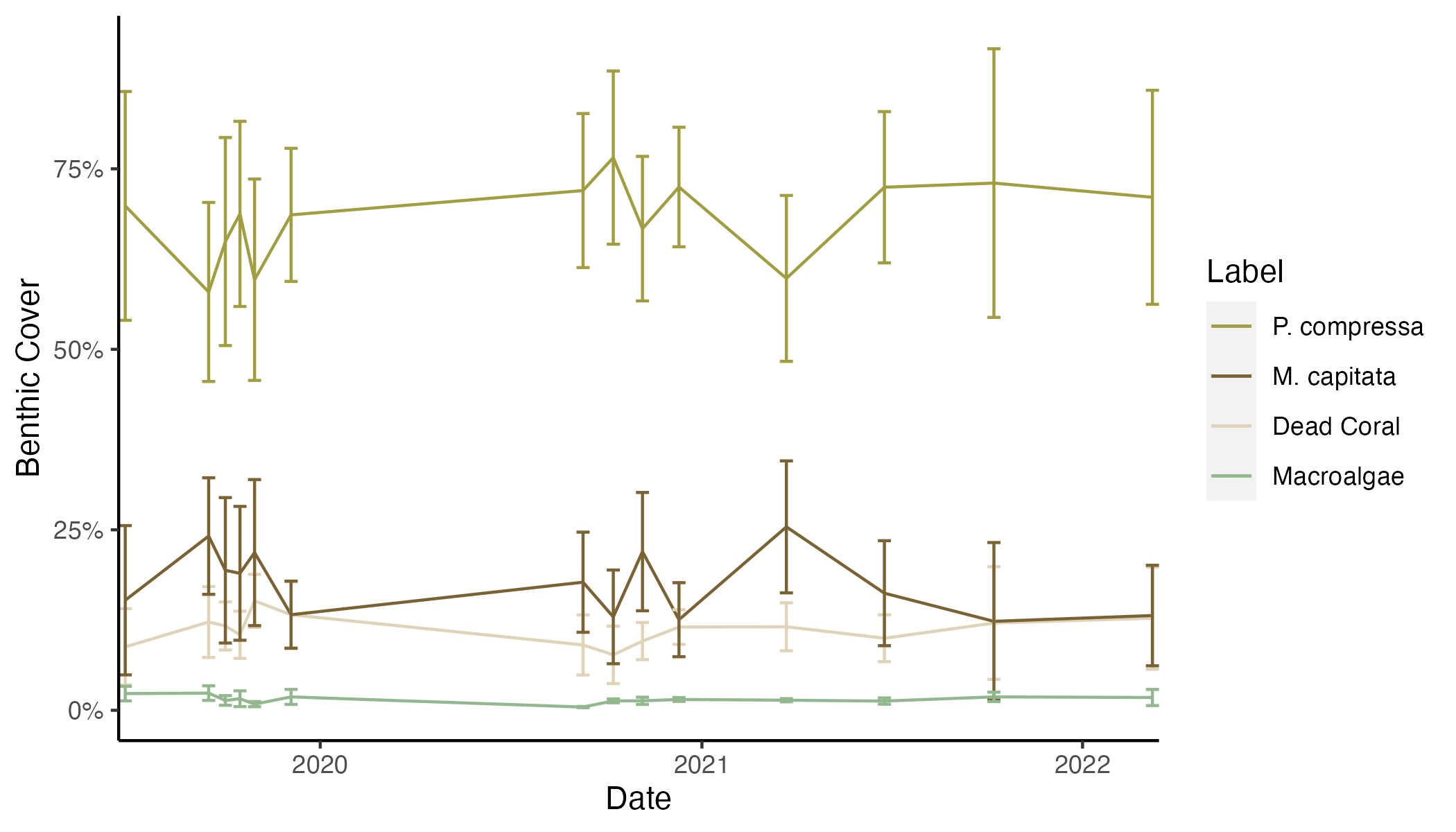

Supplement: Supplemental Information 3 — Each line represents a different species or functional group from transect analysis with means and 95% confidence intervals (transect N = 2–4) expressed as percent cover. Benthic images were taken before (June 2019), during (October 2019), and two years after (March 2022) the 2019 marine heatwave. No significant changes were observed within each species functional group. [file peerj-13-18654-s003.png]
